# Rapid review of decision-making for place of care and death in older people: Lessons for COVID-19

**DOI:** 10.1192/j.eurpsy.2021.732

**Published:** 2021-08-13

**Authors:** E. West, K. Moore, N. Kupeli, E. Sampson, P. Nair, N. Aker, N. Davies

**Affiliations:** 1 Division Of Psychiatry, University College London, Marie Curie Palliative Care Research Society, London, United Kingdom; 2 Research Department Of Primary Care And Population Health, Centre for ageing population Studies, London, United Kingdom

**Keywords:** Decision-making, COVID-19, Place of Care / Place of Death, Advance Care Planning

## Abstract

**Introduction:**

The coronavirus pandemic (COVID-19) has affected the functioning and capacity of healthcare systems worldwide. COVID-19 has also disproportionately affected older adults, including those living with dementia. In the context of COVID-19, decision-making surrounding place of care and place of death in this population involves significant new challenges.

**Objectives:**

To explore key factors that influence place of care and place of death decisions in older adults. A secondary aim was to investigate key factors that influence the process and outcome of these decisions in older adults. To apply findings from current evidence to the context of COVID-19.

**Methods:**

Rapid review of reviews, undertaken using WHO guidance for rapid reviews. Ten papers were included for full data extraction. These papers were published between 2005-2020. Data extracted was synthesised using narrative synthesis, with thematic analysis and tabulation.

**Results:**

Papers included discussed actual place of death, as well as preferred. Results were divided into papers that explored the process of decision-making, and those that explored decision-making outcomes. Factors such as caregiver capacity, the availability of multidisciplinary teams, cultural appropriateness of care packages and advanced care planning were found to be key.

**Conclusions:**

The process and outcomes of decision-making for older people are affected by many factors – all of which have the potential to influence both patients and caregivers experience of illness and dying. Within the context of COVID-19, such decisions may have to be made rapidly and be reflexive to changing needs of systems and of families and patients.

